# Association of the C-Reactive Protein Gene (*CRP*) rs1205 C>T Polymorphism with Aortic Valve Calcification in Patients with Aortic Stenosis

**DOI:** 10.3390/ijms161023745

**Published:** 2015-10-09

**Authors:** Ewa Wypasek, Daniel P. Potaczek, Anetta Undas

**Affiliations:** 1 Institute of Cardiology, School of Medicine, Jagiellonian University, 31-202 Cracow, Poland; E-Mail: mmundas@cyf-kr.edu.pl; 2 John Paul II Hospital, 31-202 Cracow, Poland; E-Mail: potaczek@staff.uni-marburg.de; 3 Institute of Laboratory Medicine, Philipps-Universität Marburg, 35043 Marburg, Germany

**Keywords:** CRP, *CRP* rs1205 C>T polymorphism, aortic valve stenosis, calcification

## Abstract

Elevation in C-reactive protein (CRP) levels have been shown in patients with aortic valve stenosis (AS). Minor allele of the CRP gene (*CRP*) rs1205 C>T polymorphism has been associated with lower plasma CRP concentrations in cohorts of healthy and atherosclerotic patients. Considering the existing similarities between atherosclerosis and AS, we examined the effect of *CRP* rs1205 C>T polymorphism on the AS severity. Three hundred consecutive Caucasian patients diagnosed with AS were genotyped for the rs1205 C>T polymorphism using the TaqMan assay. Severity of the AS was assessed using transthoracic echocardiography. The degree of calcification was analyzed semi-quantitatively. Carriers of the rs1205 T allele were characterized by elevated serum CRP levels (2.53 (1.51–3.96) *vs.* 1.68 (0.98–2.90) mg/L, *p* < 0.001) and a higher proportion of the severe aortic valve calcification (70.4% *vs.* 55.1%, *p* = 0.01) compared with major homozygotes. The effect of *CRP* rs1205 polymorphism on CRP levels is opposite in AS-affected than in unaffected subjects, suggesting existence of a disease-specific molecular regulatory mechanism. Furthermore, rs1205 variant allele predisposes to larger aortic valve calcification, potentially being a novel genetic risk marker of disease progression.

## 1. Introduction

Aortic stenosis (AS) is currently the most commonly acquired valvular heart disease in developed countries. Recent studies have provided some evidence that the pathomechanisms of atherosclerosis and AS may be partly similar and include endothelial damage, accumulation of oxidized low-density lipoproteins, infiltration of monocytes, mast cells and T lymphocytes associated with an activation of local and systemic inflammation [1,2,3]. However, the final step in the atherosclerosis process is the plaque formation in the intima of the blood vessels, while in AS severe calcification of the aortic valve represents the end-stage of the disease [4,5]. Enhanced fibro-calcification of the valve leaflets limits their mobility and causes stenosis, which leads to high pressure gradients through the aortic valve.

C-reactive protein (CRP) is a biomarker of inflammation with predictive value for cardiac events in both, apparently healthy subjects and patients with coronary artery disease (CAD), a cardiac manifestation of atherosclerosis [6]. Several studies [7,8,9], although not all [10,11], found an association between plasma CRP levels and severity and/or progression of AS. Elevated CRP levels have been reported in patients with severe symptomatic AS awaiting valve surgery [9] which were declining after aortic valve replacement [7]. In asymptomatic patients, a rapid increase in AS severity has been associated with elevated CRP levels, suggesting that CRP may be a marker of AS progression [12].

The mechanisms underlying the association between plasma CRP and AS, in particular the role of genetic factors, are still unclear. However, much is known about the general contribution of genetic determinants to plasma CRP levels regulation. Large genetic association studies of a various types, including genome-wide association studies (GWAS), have demonstrated that a substantial portion of the inter-individual variability in inflammatory biomarkers including CRP is genetically determined [13,14,15,16,17,18]. One of the polymorphism showing the most uniform and consistent association with CRP levels is CRP gene (*CRP*) rs1205 C>T variant. A cross-sectional population-based Genetics of Lipid Lowering Drugs and Diet Network (GOLDN) study has demonstrated the minor allele carriage of *CRP* rs1205 polymorphism to be associated with lower CRP levels in a total of 1123 white US participants [13]. Similarly, analyses of a total 2523 either healthy or community-based Caucasians representing three independent cohorts, namely the Women’s Health Study (WHS), the Physician’s Health Study (PHS), and the study of Pravastatin Inflammation/CRP Evaluation (PRINCE), have consistently shown that the minor allele of rs1205 polymorphism was in all three cases associated with lower CRP concentrations [14]. Also, in two independent cohorts of the population-based Cardiovascular Health Study (CHS) comprising either 3941 white European American and 700 African American participants, the minor allele of rs1205 polymorphism was consistently associated with lower plasma CRP levels [15]. Those findings were corroborated by several GWAS on CRP showing the rs1205 variant itself or its linkage disequilibrium (LD) proxies to give a peak association signal [16,17,18,19].

Considering all these facts, we speculated that the minor allele of *CRP* rs1205 polymorphism can be associated with decreased CRP levels in subjects with AS and thus also with the less severe disease in this group of patients. To verify those hypotheses, the rs1205 polymorphism was genotyped in three hundred individuals suffering from AS, in whom plasma CRP concentrations were measured and the severity of AS was assessed. To the best of our knowledge, the effects of *CRP* rs1205 polymorphism in AS have not been studied so far.

## 2. Results

The age of 300 subjects with AS included in the study ranged from 22.0 to 84.0 years with a median of 66.0 years. One hundred sixty-one (53.7%) patients were male. The genotype distribution of *CRP* rs1205 C>T polymorphism was as follows: CC, 109 (36.3%), CT, 152 (50.7%) and TT, 39 (13.0%), showing no deviation from Hardy-Weinberg Equilibrium (HWE; *p* = 0.46). The T allele frequency of 38.3% was not much higher than in the previous Caucasian population- or healthy subjects-based studies on the rs1205 polymorphisms, in which it reached a value of 32.5% [13], 34.4% [20] or 34.2% [15].

Nevertheless, to additionally validate the genotyping method, it was also applied in an independent group of 84 healthy subjects aged between 19 and 58 years (median of 37.5 years) and comprising 24 males (28.6%), in whom the following genotype distribution was obtained: CC, 33 (39.3%), CT, 44 (52.4%) and TT, 7 (8.3%), also being in HWE (*p* = 0.35). The rs1205 polymorphism minor allele frequency of 34.5% was completely in line with those reached in community- or healthy individuals-based studies conducted in subjects of European descent and given above [13,14,15]. Although our set of healthy subjects cannot be considered a control group to our main study cohort of AS patients, the genotype distribution and allele count were compared between the groups both showing no significant differences (*p* = 0.50 and *p* = 0.37, respectively).

### 2.1. Association with CRP Levels

**Figure 1:**
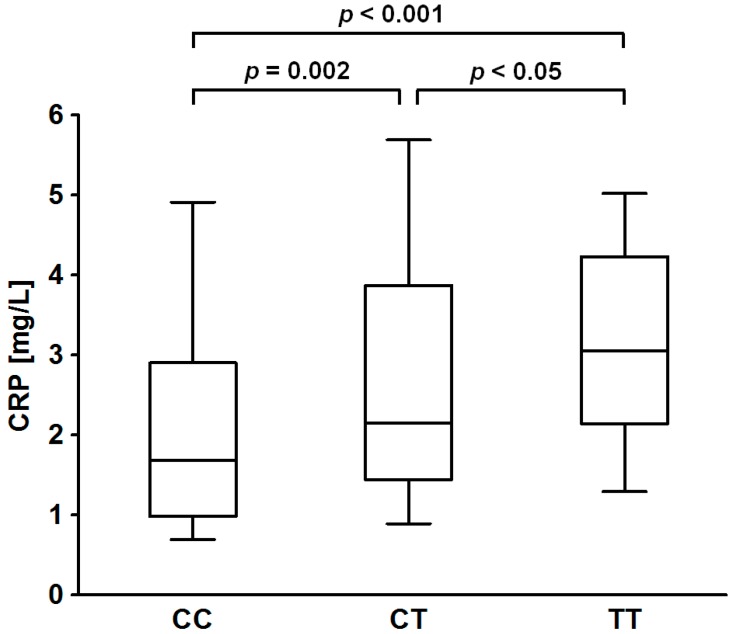
Boxplot describing the relationship of plasma C-reactive protein (*CRP*) gene rs1205 C>T polymorphism with the CRP levels; boxes range from the 25th to the 75th percentile with a horizontal black line at the median and vertical lines extending to the 10th and 90th percentiles. Crude *p*-values calculated by Mann-Whitney *U* test are given.

In our main study cohort of 300 AS patients, of the laboratory parameters of interest, *i.e.*, fibrinogen and CRP, only the latter demonstrated a significant association with *CRP* rs1205 C>T polymorphism in a genotype model; CRP levels were the highest in minor homozygotes and the lowest in subjects carrying two major alleles (3.05 (2.14–4.23) *vs.* 2.14 (1.44–3.87) *vs.* 1.68 (0.98–2.90) mg/L; *p* < 0.001) (Table 1, Figure 1). Furthermore, linear regression showed rs1205 polymorphism to be associated with CRP concentrations also in an additive model, which remained strongly significant after adjustment for demographics, accompanying disorders and drugs (Table 2). Similar results were obtained when the effect of the polymorphism on CRP levels was analyzed in a dominant model (CRP concentration in patients carrying the minor allele, 2.53 (1.51–3.96) mg/L; *p* < 0.001), also when assessed by linear regression irrespectively of the adjustment for potential confounders (Table 2).

On the contrary, in our independent group of 84 healthy individuals, CRP levels were the lowest in minor homozygotes and the highest in carriers of the two major alleles (0.36 (0.34–0.57) *vs.* 0.58 (0.39–1.38) *vs.* 1.03 (0.56–1.86) mg/L; *p* = 0.02, for a genotype model), which was confirmed by a linear regression analysis in an additive model (*p* = 0.01), also including the adjustment for age, sex, body mass index and smoking status (*p* = 0.01). Similar association was observed in a dominant model (CRP levels in the minor allele carriers, 0.57 (0.36–1.34) mg/L; *p* = 0.03), also when analyzed by linear regression without (*p* = 0.04) and with the analogous adjustment (*p* = 0.02).

**Table 1:** Demographic, clinical and laboratory characteristics of 300 aortic valve stenosis patients with regard to the C-reactive protein (*CRP*) gene rs1205 C>T polymorphism.

	Major (CC) Homozygotes, *n* = 109	Heterozygotes (CT), *n* = 152	Minor (TT) Homozygotes, *n* = 39	*p*-Value
**Demographic and Clinical Characteristics**				
Age, years	67.0 (60.0–73.0)	66.0 (58.0–72.0)	65.0 (58.0–73.0)	0.78
Males, *n* (%)	61 (56.0)	81 (53.3)	19 (48.7)	0.73
Body mass index, kg/m^2^	26.7 (24.3–29.6)	27.1 (24.8–30.1)	28.6 (26.8–30.4)	0.06
Current smokers, *n* (%)	15 (13.8)	19 (12.5)	7 (17.9)	0.68
Hypertension, *n* (%)	71 (65.1)	92 (60.5)	23 (59.0)	0.69
Diabetes mellitus, *n* (%)	23 (21.5), *n* * = 107	31 (20.4)	6 (16.2), *n* = 37	0.79
**Medications**				
β-blockers, *n* (%)	49 (44.9)	75 (49.7), *n* = 151	16 (41.0)	0.56
ACE inhibitors, *n* (%)	49 (44.9)	75 (49.3)	14 (35.9)	0.31
Statins, *n* (%)	56 (51.4)	76 (50.0)	15 (38.5)	0.36
Vitamin K antagonists, *n* (%)	15 (13.8)	25 (16.4)	7 (17.9)	0.77
**Echocardiographic Parameters**				
Mean transvalvular gradient, mmHg	43.9 (33.5–59.4), *n* = 107	51.3 (38.8–66.7)	56.6 (40.0–68.0), *n* = 37	0.09
Maximal transvalvular gradient, mmHg	77.3 (59.0–100.0)	85.8 (59.4–104.5)	90.0 (69.4–105.0)	0.17
Ejection fraction, %	64.0 (56.0–69.3)	65.0 (57.3–68.4)	64.7 (55.0–70.0)	0.71
Aortic bulb, mm	37.0 (33.0–40.0), *n* = 90	37.0 (33.0–40.0), *n* = 120	37.0 (31.0–42.0), *n* = 30	0.87
Ascending aorta, mm	38.0 (34.0–41.0), *n* = 98	38.0 (33.0–42.0), *n* = 132	36.0 (31.0–39.0), *n* = 33	0.28
Maximum aortic jet velocity, m/s	4.24 ± 0.78, *n* = 53	4.23 ± 0.79, *n* = 67	4.65 ± 0.87, *n* = 18	0.12
Aortic valve area, cm^2^	0.80 (0.58–0.97), *n* = 108	0.70 (0.53–0.97)	0.70 (0.60–0.93)	0.37
**Severe aortic Valve Calcification, *n* (%) ^†^**	59 (55.1), *n* = 107	100 (66.7), *n* = 150	33 (84.6)	0.003 ‡
**Laboratory Measurements**				
Creatinine, mmol/L	82.0 (69.0–97.0)	79.0 (65.9–93.0)	77.0 (69.2–87.0)	0.23
Glucose, mmol/L	5.30 (4.70–5.80)	5.20 (4.90–5.95)	5.20 (4.90–5.80)	0.71
Total cholesterol, mmol/L	4.55 (3.58–5.59)	4.55 (3.47–5.46)	4.51 (3.62–5.87), *n* = 38	0.93
Low-density lipoprotein cholesterol, mmol/L	2.89 (2.10–3.58)	2.83 (2.01–3.68)	2.82 (2.34–3.73), *n* = 38	0.87
High-density lipoprotein cholesterol, mmol/L	1.30 (1.08–1.55)	1.28 (1.02–1.52)	1.29 (1.08–1.59), *n* = 38	0.45
Triglycerides, mmol/L	1.17 (0.88–1.80)	1.39 (1.06–1.85)	1.29 (0.90–1.69), *n* = 38	0.08
Fibrinogen, g/L	3.26 ± 1.13	3.34 ± 1.09	3.70 ± 1.07	0.10
CRP, mg/L	1.68 (0.98–2.90), *n* = 107	2.14 (1.44–3.87)	3.05 (2.14–4.23), *n* = 37	<0.001 ^‡^

Quantitative data are given as mean ± standard deviation or median (interquartile range). *p*-Values were obtained using one-way ANOVA, Kruskal-Wallis ANOVA, or Pearson’s χ^2^ test, as appropriate. ACE denotes angiotensin-converting enzyme; * Number (*n*) of subjects with a certain genotype having relevant data (if different from the total number of subjects with that genotype); **^†^** Subjects were considered as having severe aortic valve calcifications if their semi-quantitative grade of 2 (large calcifications) or 3 (massive calcium deposits) was detected by transthoracic echocardiography, while the remaining subjects were classified as having mild calcifications; ^‡^ Significant after correction for multiple testing (nominal significance level = 0.05; significance level after correction for multiple testing = 0.005; for details, please, refer to the main text, Section 4.6.).

**Table 2:** The effect of C-reactive protein (*CRP*) gene rs1205 C>T polymorphism on CRP levels analyzed by (multiple) linear regression.

Genotype Groups (Ordered from Left to Right)	Crude		Adjusted *		Adjusted ^†^	
	β (SE)	*p*-Value	β (SE)	*p*-Value	β (SE)	*p*-Value
CC/CT/TT	0.25 (0.06)	<0.001	0.20 (0.06)	<0.001	0.20 (0.06)	<0.001
CC/CT + TT	0.22 (0.06)	<0.001	0.19 (0.06)	<0.001	0.18 (0.06)	0.002

β denotes partial correlation coefficient; SE denotes standard error; * For age, sex, body mass index, smoking status, diabetes, hypertension and drugs (β-blockers, angiotensin-converting enzyme inhibitors, statins and vitamin K antagonists); **^†^** For the variables listed above (“*”) plus aortic valve calcifications classified as described in the legend “**^†^**” of Table 1.

Thus, the association between *CRP* rs1205 variant and plasma CRP concentrations present in our healthy subjects is directly in line with those reported by previous population- or healthy individuals-based studies [13,14,15], whereas its direction we observed in AS subjects is quite opposite.

### 2.2. Association with AS Severity

**Figure 2:**
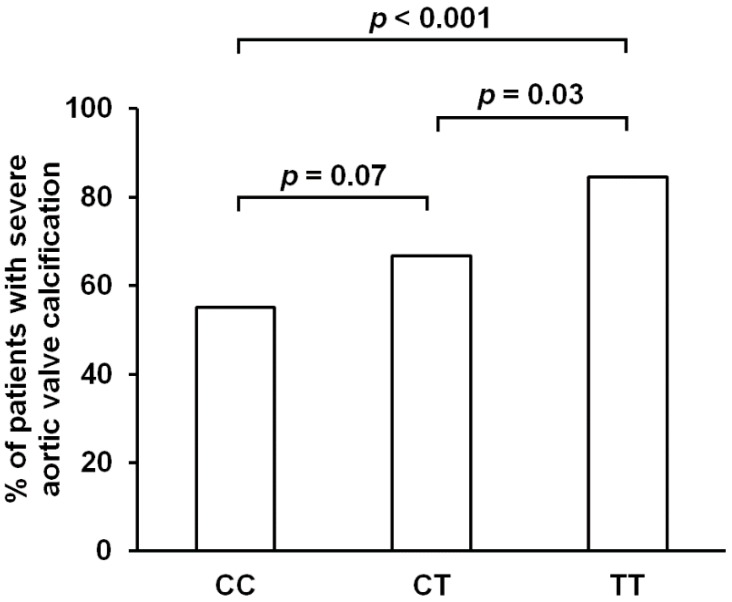
Percentage of patients with severe aortic valve calcification with regard to the genotype of plasma C-reactive protein (*CRP*) gene rs1205 C>T polymorphism. Crude *p*-values calculated by two-tailed Fisher’s exact test are given.

In a genotype model, *CRP* rs1205 C>T polymorphism turned out to be associated with severe aortic valve calcification; the highest rate of subjects with severely calcified valve was present among minor homozygotes and the lowest in subjects possessing two major alleles (84.6% *vs.* 66.7% *vs.* 55.1%; *p* = 0.003) (Table 1, Figure 2). Also in patients carrying the minor allele, the percentage of those with severe aortic valve calcification (70.4%) was higher when compared to major homozygotes (*p* = 0.01). Further analyses conducted by logistic regression demonstrated that the chance of having severe aortic valve calcifications was substantially and significantly higher in minor homozygotes when compared to subjects with two major alleles, also after adjustment for demographic parameters, concomitant diseases and medications (Table 3). Contrasting heterozygotes *vs.* major homozygotes showed a similar effect of lower odds in the latter to be present, which was however reaching only a statistical tendency or marginal significance depending on the adjustments (Table 3). In addition, also the analysis in a dominant model gave consistent results; the odds of having severely calcified aortic valve were, irrespective of the adjustments applied, almost two times higher in carriers of the minor allele when compared to the remainders (Table 3). Of note, inclusion of CRP levels in the adjustment analyses did not influence the effects of the polymorphism on the chances of having severe valve calcification (Table 3).

**Table 3:** The effect of C-reactive protein (*CRP*) gene rs1205 C>T polymorphism on the risk of severe aortic valve calcification * analyzed by logistic regression.

Genotype Groups	Crude		Adjusted ^†^		Adjusted ^‡^	
	OR (95% CI)	*p*-Value	OR (95% CI)	*p*-Value	OR (95% CI)	*p*-Value
**CC (reference)/CT/TT**						
CT *vs.* CC	1.63 (0.98–2.71)	0.06	1.70 (1.01–2.86)	0.047	1.72 (1.01–2.92)	0.04
TT *vs.* CC	4.47 (1.73–11.57)	0.002	4.14 (1.57–10.96)	0.004	4.25 (1.59–11.37)	0.004
**CC (reference)/CT + TT**						
CT + TT *vs.* CC	1.93 (1.18–3.16)	0.009	1.95 (1.18–3.24)	0.009	1.97 (1.18–3.30)	0.01

OR denotes odds ratio; CI, confidence interval; * As described in the legend “**^†^**” of Table 1. **^†^** For the variables listed in the legend “*” of Table 2; **^‡^** For the variables listed in legend “*” of Table 2 plus C-reactive protein levels.

To better understand this part of our results, we also calculated pairwise correlations between the two variables associated with rs1205 polymorphism. Increase in CRP levels did not correlate with severe calcification (*r* = 0.04, *p* = 0.50). These together with the mutual adjustments conducted for severe calcification (Table 2) or CRP concentrations (Table 3) show that the associations between rs1205 and CRP levels or advanced calcifications are independent of each other. Additionally, no associations of CRP polymorphism with the echocardiographic parameters were found.

## 3. Discussion

For the first time, we show that AS patients carrying the rs1205T allele are characterized by more severe aortic valve calcification. We also demonstrate here that the possession of *CRP* rs1205 C>T polymorphism minor allele is associated with elevated CRP levels in AS patients. A completely opposite effect of the rs1205 substitution with its C allele driving an increase in CRP concentrations has been demonstrated by previous studies [13,14,15]. Although it could have been theoretically possible that the association we found in AS patients appeared accidentally, it was really difficult for us to believe in that, considering its magnitude and significance which are both resistant to extensive adjustments. Thus, together with the fact that the previous studies have analyzed the effect of the rs1205 on plasma CRP concentrations in population- or healthy individuals-based cohorts [13,14,15] and that we did it in a highly specific selection of AS subjects, it made an alternative hypothesis also possible, *i.e.*, that being completely opposite to that reported for (at least in a huge majority) AS-unaffected subjects the effect observed by us in AS patients is disease-specific. To verify this speculation, we decided to check how the relationship between the rs1205 polymorphism and plasma CRP levels would look like in an independent, though limited in size, group of healthy subjects of the same ethnical background, in whom genotyping and CRP assessment would be carried out with exactly the same methods as in AS patients. Interestingly enough, even in this cohort of unaffected individuals, a clear association between the rs1205 and CRP concentrations resistant to adjustments was present in both models tested, which direction was exactly in line with the previous large community- or healthy subjects-based studies [13,14,15], supporting the above mentioned hypothesis of AS-specific effect of rs1205 on CRP levels.

Hence, it is probable that rs1205 polymorphism acts as a sort of molecular switch of CRP synthesis, differentially influenced by distinct molecular environments in AS-affected (and perhaps susceptible) and healthy/AS-free subjects. The mechanisms potentially underlying this complex regulation remain however to be established. The most interesting option would be the role of rs1205 polymorphism related to its location in 3ʹ-untranslated region (3ʹ-UTR) of *CRP*. Polymorphisms of this region are known for their potential to affect mRNA stability and thus expression of gene harboring them [21,22]. Interestingly, 3ʹ-UTR of *CRP* is disproportionally long suggesting its special regulatory importance [23]. The effect of 3ʹ-UTR polymorphism on mRNA stability can by exerted in several ways, for example by changing the sequence of AU-rich elements belonging to the most important determinants of mRNA stability [24,25], in particular in cytokine genes [23,24]. Although rs1205 C>T substitution creates an AU motif on the mRNA level, the manual assessment of the remaining sequence surrounding the polymorphism suggests it not to be a case of a real AU-rich motif. This is confirmed by the *in silico* analysis of the rs1205 polymorphisms within its closest mRNA neighborhood (±30 nt), conducted using RegRNA 2.0 software (http://regrna2.mbc.nctu.edu.tw/) [26], showing that no AU-rich elements are present in the region independently of the polymorphism. Another option for rs1205 polymorphism to functionally affect *CRP* mRNA stability could theoretically be its eventual effect on microRNAs (miRNA) binding sites, another element of posttranscriptional regulation of gene expression, for which 3ʹ-UTR is thought to be the main target of [27]. AS patients have distinctive miRNA profiles [28] making it possible that this type of *CRP* gene expression control differs between AS or AS-predisposed individuals and healthy (or non-AS-affected or -predisposed) subjects, which could potentially provide a functional support for our association findings. However, bioinformatic analysis by RegRNA 2.0 software demonstrates that no miRNA target motifs are present in the selected region, again independently of the polymorphism [26]. Then, a less fancy but quite probable option would be that rs1205 polymorphism influences *CRP* expression by its LD with another, very functional genetic variant, which is a rather frequent situation [22,29]. Using a proxy search function of SNAP software (https://www.broadinstitute.org/mpg/snap/ldsearch.php) [30] to screen 1000 Genomes Pilot 1 CEU population data [31] for polymorphisms being with rs1205 in a high LD (*r*^2^ > 0.8) and at a distance not longer than 500 kb, eight variants can be found (effective *r*^2^ between 0.96 and 1.0) of which three are located downstream (rs2808628, rs2808629 and rs2794520) and five within 15 kb upstream (rs2027471, rs1341665, rs7551731, rs7553007 and rs4546916) of *CRE*. If *in silico* analyzed (AliBaba 2.1 program using TRANSFAC 4.0-based matrices; http://www.gene-regulation.com/pub/programs/alibaba2/index.html) [32], the first four of the latter five polymorphisms show an ability to affect binding of transcription factors (data not shown), and thus could potentially influence CRP expression by an altered promoter activity. Similarly to miRNA, one might speculate that different transcription factors can be expressed in AS/AS-susceptible and in unaffected/non-predisposed individuals making putative effects of any polymorphism being in high LD with rs1205 on *CRP* transcription varying between these groups of subjects. Interestingly, polymorphisms belonging to rs1205 tagging bin, such as rs1341665, rs2027471, rs7553007, rs2794520, rs2808628, rs2808629 and rs1205 itself, gave in a recent Sardinian population-based GWAS a peak CRP levels association signal [16] consistent in its direction with the effects observed in previous candidate-gene studies [13,14,15] and our small group of healthy individuals. Furthermore, an association between rs7553007 polymorphism and CRP concentrations was found to be a top hit also in a large GWAS by Elliott *et al.* [17] comprising several community-based and cardiologic or metabolic case-control cohorts of European or Indian Asian ethnicity. The direction of the effect was again consistent with the previous studies [6,13,14,15,16,17,18,33,34].

It is also possible that the complex LD/haplotypic relationships can functionally underlie the effects of the rs1205 polymorphism on CRP synthesis, for example by its interaction with rs2808630 and (potentially) functional *CRP* promoter rs3091244 variants [19,35]. Similar complex LD/haplotypic interplay might possibly underlie another finding of our study. In addition to its association with CRP levels, we detected a strong association between minor allele of rs1205 polymorphism and more severe aortic valve calcification. Interestingly enough, those two associations were independent from each other, which made it impossible to state that it is (an association of rs1205 with) CRP that drives (the effect of rs1205 on) an increase in aortic valve calcification status, although such an explanation would be really tempting due to previous laboratory and clinical findings suggesting that an elevation in CRP levels (and inflammation) can correlate with an increase in aortic wall or valve calcification [36,37]. Thus, at this point it is not easy to explain the observed association of rs1205 polymorphism with aortic valve calcification. Indeed, as stated at the beginning of this paragraph, it may result from a possibly complex LD/haplotypic interplay between rs1205 and one or several polymorphisms in *CRP* neighboring regions of chromosome 1q23, a locus harboring many inflammation-related and -susceptibility genes [16,19,38], products of which could functionally affect the calcification process, although it would be difficult to suggest any obvious candidates. For example, a polymorphism rs3845624 located halfway between *DARC* and *FCER1A*, *i.e.*, about 405 kb upstream of *CRP*, was found in GWAS to be a source of an association with CRP levels, independent of the signals from *CRP* and thus not possible to be explained by simple LD with them (only) [16,38]. Hence, by analogy, one could speculate that also the effect on calcification observed for rs1205 could be functionally underlain by complex, even partially weak, linkage disequilibrium (LD)/haplotypic interaction with polymorphisms located in other inflammatory genes of 1q23 locus. These speculations might be supported from the studies on a potential role of *CRP* polymorphisms, including an rs1205, and the risk of atherosclerosis-related disorders. *CRP* variants related to CRP levels demonstrated none or inverted (the case of rs1205) association with MI or ischemic stroke, while one variant not influencing CRP concentrations affected the risk of atherothrombotic events in the earlier study by Miller *et al.* [14]. Subsequently, a huge mendelian randomization meta-analysis by Wensley and colleagues [39] clearly showed that CRP levels genetically determined by *CRP* polymorphisms including rs1205 are unrelated to CAD risk. Likewise, in AS the effect of rs1205 on calcification is likely to be mediated by one of the mechanisms we suggest above rather than through its influence on CRP concentrations.

This study has several limitations. First, our group of healthy subjects was rather small but it was not used as a main study cohort or its control group but rather for confirmation of the accuracy of the genotyping methodology and the direction of the previously reported association between rs1205 variant and CRP levels in AS-unaffected subjects; Second, because we focused on the polymorphism that represents already identified major tagging bin influencing CRP levels, *CRP* fine-mapping was not performed, which could be however useful considering the direction of the association observed in AS and thus should be possibly conducted in a bigger cohort in future; Third, we did not conduct confirmatory analysis of the association between rs1205 polymorphism and CRP concentrations or calcification in a replication cohort but we would prefer them to be addressed by an independent group in a separate study, so that no “combined” analyses with potential bias of the results would be tempting; Fourth, we did not measure calcification of aortic valves by computed tomography, which is a more precise method compared with echocardiography. Also, since the association between *CRP* rs1205 polymorphism and aortic valve calcification was not accompanied by similar findings for echocardiographic parameters, our observation should be followed by studies analyzing this phenomenon, including those applying a more accurate estimation of the calcium amount in the valves by means of micro CT scanning.

To summarize, our findings suggest that the minor allele of the rs1205 *CRP* polymorphism could serve as a potential marker identifying subjects prone to develop severe and heavily calcified AS. This genetic variant might possibly be a risk marker of AS progression.

## 4. Materials and Methods

### 4.1. Patients

A total of 312 consecutive patients with acquired AS referred to either Cardiology Clinic, John Paul II Hospital or the Department of Cardiovascular Surgery and Transplantation, Jagiellonian University School of Medicine, both in Cracow, Poland for further diagnostic work-up between September 2009 and May 2012 were recruited. Of those, 12 subjects were ineligible due to incomplete clinical and laboratory data (*n* = 10) or patient refusal (*n* = 2); thus 300 patients were available for the purposes of the present study. All patients came from Malopolska region in the South of Poland.

The exclusion criteria were: acute infection, Valsalva sinus aneurysm or rheumatic AS, known cancer, autoimmune disorders, endocarditis, previous cardiac surgery, and a history of myocardial infarction (MI), stroke, venous thromboembolism or bleeding. Smoking was defined as the use of 1 or more cigarettes per day. Patients receiving insulin or oral hypoglycemic agents or having random fasting glucose levels of >7 mmol/L at least twice were classified as having diabetes mellitus. Arterial hypertension was diagnosed based on a history of hypertension (blood pressure >140/90 mmHg) or preadmission antihypertensive treatment.

The Jagiellonian University Ethical Committee approved the study, and all the participants provided their written informed consent.

### 4.2. Echocardiography

Transthoracic echocardiography was performed in each patient using a MargotMac 5000 ultrasound machine with conventional techniques corresponding to the European Society of Cardiology guidelines. The aortic valve area (AVA) was calculated using the standard continuity equation [40]. The transvalvular gradient was measured by Doppler echocardiography using the modified Bernoulli equation [40].

### 4.3. Valvular Calcification

Diseased aortic valves were collected during surgery for valve replacement. Valvular calcification was analyzed semi-quantitatively by macroscopic evaluation as described previously [41]. The following scoring system was adopted: 0, absence, 1, fine calcifications (isolated calcium deposits), 2, large calcifications (calcification occupying less than 20% of the total leaflet area), and 3, abundant calcium deposits (occupying at least or more than 20% of the total leaflet area). In this study, grades 0 and 1 were considered mild, while 2 and 3 were considered to be severe calcifications.

### 4.4. Laboratory Tests

Fasting venous blood was drawn from each patient, between 7 and 9 AM. Citrated blood samples (9:1 of 0.106 M trisodium citrate) were centrifuged at 2500× *g* at 4 °C for 20 min, then stored in aliquots at −80 °C until analysis. Lipid profile, glucose and creatinine were assayed by routine laboratory techniques. Fibrinogen was determined using the Clauss method. High-sensitivity CRP was measured by immunoturbidimetry (Roche Diagnostics GmbH, Mannheim, Germany).

### 4.5. Genotyping of CRP Polymorphism

DNA was extracted from whole blood or a buffy coat using the NucleoSpin^®^ Blood DNA Kit (Macherey-Nagel GmbH & Co KG, Düren, Germany) according to the manufacturer’s protocol and stored at −80 °C until analysis. Genotypes of *CRP* rs1205 C>T polymorphism (previously reported as: 1846 G>A (13), 3872 G>A (14) or 3u2131C>T (12)) were ascertained by the allelic discrimination test using TaqMan Genotyping assay on the ABI PRISM 7900HT Fast Real-Time PCR System (Life Technologies Co., Carlsbad, CA, USA; assay ID: C_7479334_10, Life Technologies Co.).

To further verify the correctness of the genotyping methodology and performance, we analyzed the distribution of the genotypes in an independent group of 84 healthy individuals of the same ethnical background in the same technical settings. The exclusion criteria were: personal and/or family history of cardiovascular diseases e.g., venous thromboembolism, myocardial infarction, angina, heart failure, sudden death, stroke, and any of chronic diseases. Although for different purposes, CRP levels were also measured in this group.

### 4.6. Statistical Analysis

The distribution of the quantitative variables was analyzed by Shapiro-Wilk test. Those normally distributed were compared using one-way ANOVA or Student’s *t* test and are presented as mean ± standard deviation. Variables deviating from the normal distribution were analyzed by Kruskal-Wallis ANOVA or Mann-Whitney *U* test and are presented as median (interquartile range) if not otherwise indicated. Linear regression was used to analyze crude and adjusted effects of the polymorphism on CRP levels with a partial correlation coefficient (standard error) as an effect measure. If necessary, the distribution of the variable entering the model was approximated to the normality using either squared or natural logarithm transformation, as appropriate. Qualitative parameters were analyzed by Pearson’s χ^2^ or two-tailed Fisher’s exact test. Crude and adjusted logistic regression was applied to assess the effects of the genetic variant on the risk of severe aortic valve calcification with odds ratio (95% confidence interval) as an output. Pairwise correlations were calculated using Pearson coefficient on variables approximated to the normal distribution if required. A value of less than 0.05 was considered statistically significant. Moreover, a level of statistical significance corrected for multiple testing was also established for our basic calculation in a genotype model given in Table 1 (CC *vs.* CT *vs.* TT). Briefly, an initial significance value of 0.05 was divided by the product of number of polymorphisms (*n* = 1) and number of dependent variables of interest (*n* = 10; CRP and fibrinogen levels, severe aortic valve calcification and 7 echocardiography parameters given in Table 1) resulting in a value of 0.005. Therefore, a *p*-value of less than 0.005 in a genotype model were a prerequisite for any further testing, which was a case of severe aortic valve calcification and CRP concentration. Statistical calculations were performed using STATISTICA Version 10.0 (StatSoft, Inc., Tulsa, OK, USA) or MedCalc Version 13 (MedCalc Software bvba, Ostend, Belgium) program.
